# Is More Always Better? A Randomized Comparative Clinical Trial About the Impact of Polydioxanone Threads Quantity for Facial Lifting

**DOI:** 10.1093/asjof/ojaf002

**Published:** 2025-01-11

**Authors:** Marcelo Germani, Victor R M Munoz-Lora, Ana C N Carnevali, Adriana Marques Geroldo, Fernanda Fogolin Teixeira, Gabriela Giro

## Abstract

**Background:**

Minimally invasive aesthetic procedures, such as the use of polydioxanone (PDO) threads, are increasingly popular for facial rejuvenation.

**Objectives:**

This study investigates the impact of the number of PDO threads on tissue displacement, volume changes, and patient satisfaction.

**Methods:**

This randomized controlled trial involved 22 patients seeking facial lifting using PDO threads. Participants were divided into 2 groups: G1 with 3 threads per hemiface and G2 with 6 threads per hemiface. Three-dimensional stereophotogrammetry was used to evaluate volumetric changes and tissue displacement at baseline, 20 days, and 60 days posttreatment. Patient satisfaction was assessed using the Global Aesthetic Improvement Scale (GAIS).

**Results:**

Significant volumetric changes were observed over time in both midface and lower face regions (*P* < .05), but no significant intergroup differences were found (*P* > .6). Tissue displacement showed statistical significance over time (*P* = .039) but not between groups (*P* = .821). GAIS scores did not differ significantly between groups or between patients and specialists. Adverse events were minor and transient, primarily involving pain.

**Conclusions:**

The number of PDO threads used did not significantly influence sustained lifting outcomes or patient satisfaction. Initial improvements in volume and tissue displacement diminished by 60 days, suggesting that additional threads do not enhance long-term efficacy. Further studies with longer follow-up are needed to better understand collagen stimulation’s potential role in lasting effects.

**Level of Evidence: 2 (Therapeutic):**

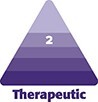

Minimally invasive aesthetic procedures have gained recognition and preference among individuals seeking facial rejuvenation without surgery. This trend is globally observed, reflecting a significant rise in demand for these kinds of treatments.^1^ Facial rejuvenation using facial threads is commonly practiced in Asia. However, data on procedures performed worldwide indicate that facial threads are not among the most popular therapies.^2^

Facial threads come in various shapes, sizes, and compositions.^3^ Polydioxanone (PDO) threads are frequently employed because of their high biocompatibility, reabsorbability, ability to stimulate collagen production, and affordability. PDO threads are completely reabsorbed by the body through hydrolysis within 4 to 6 months, although their collagen-stimulating effects may persist beyond this period.^4^ Facial soft-tissue repositioning using PDO barbed threads aims to improve the appearance of wrinkles and folds by stimulating collagen and generating a direct tensor effect, which refers to the mechanical lifting and tightening of the tissues as the barbs on the threads create traction and hold the tissues in place.^5^ This involves uplifting facial soft tissue by creating traction after inserting barbed PDO threads, which are anchored to the superficial musculoaponeurotic system (SMAS).

This approach is not new and has evolved significantly over the years. However, there is still a clear gap in the literature regarding the real efficacy and longevity of the results achieved with this technique, which may depend on the angle of insertion, location, and especially, the number of PDO threads used.^6,7^ It may seem obvious to believe that a greater number of threads will result in greater traction and tissue displacement. However, because PDO threads are anchored to nonfixed facial soft tissue, long-term results, and patient satisfaction may not be affected and sustained by the quantity used.

Therefore, this clinical study aimed to assess and compare the influence of the number of PDO threads on facial soft-tissue displacement and volume changes, as well as patient satisfaction, using quantitative and semiquantitative assessments.

## METHODS

### Study Design

This randomized controlled clinical trial obtained ethical approval from the Kaiser Clínica & Hospital Dia Research Ethics Committee, under protocol number CAAE—77996423.2.0000.028. All patients provided written informed consent prior to inclusion. The study followed guidelines for Good Clinical Practice and adhered to the Declaration of Helsinki for human investigations. The present study is registered under the trial number U1111-1317-4301, ensuring compliance with ethical standards and providing transparency in the research process.

### Study Participants

Patients of both genders, aged between 30 and 60 years, with signs of tissue ptosis because of aging and seeking facial lifting using PDO threads were recruited between June 1 and June 10, 2024, from our private practice in Lins, São Paulo, Brazil. The study duration extended 60 days postrecruitment, concluding on August 9, 2024. The patients were not compensated for their participation in this study. The treatment was provided free of charge to all participants to avoid financial burden and not to impact the satisfaction scores or reports. Participants with severe preexisting medical conditions, such as neuromuscular disorders, blood clotting disorders, known allergies to product components, active infections in the proposed treatment area, pregnant or breastfeeding individuals, those who had undergone any form of aesthetic treatment in the last 6 months, and patients with any previous history of thread application were excluded from the study. Mild and well-controlled conditions, such as hypertension or Type 2 diabetes, were not considered exclusion criteria, if they were managed appropriately and did not pose a risk to the procedure. The cutoff for inclusion/exclusion was based on the safety profile of PDO-thread treatments and potential risks associated with these conditions.

**Figure 1.**
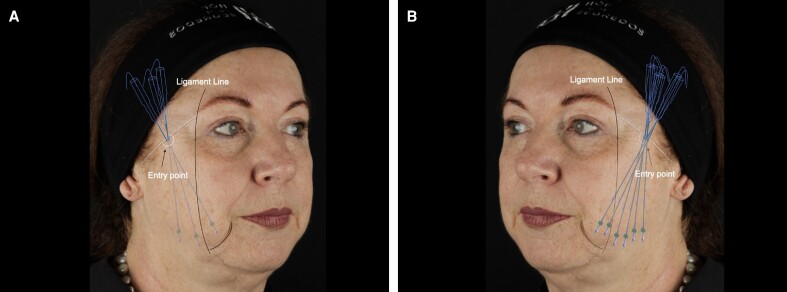
Schematic representation of the cannula entry point for (A) 6 (Group 1) and (B) 12 (Group 2) PDO-thread placement in a 54-year-old female patient seeking facial soft-tissue repositioning. Threads endpoints are positioned 1.5 and 1 cm apart in Groups 1 and 2, respectively.

The participants were divided into 2 groups (*n* = 11; Figure 1) using a random allocation software (Random Allocation Software 2.0, Mahmood Saghaei):

- Group 1 (G1): participants who received 3 PDO threads in each lateral hemiface, totaling 6 threads per participant.
- Group 2 (G2): participants who received 6 PDO threads in each lateral hemiface, totaling 12 threads per participant.

### Polydioxanone Threads Positioning Technique

All participants were instructed to wash their faces with water and soap, followed by facial asepsis using 2% alcoholic chlorhexidine (Riohex, Rioquímica, São Paulo, Brazil).

For this study, 150 mm length PDO threads (iThread, São Paulo, Brazil) with cutting barbs inserted into a 19 G 100 mm cannula were used. First, local anesthesia was applied at the cannula entry point located 10 mm above the zygomatic arch and 50 mm lateral from both eyes’ outer canthus region (Figure 1). The entry point was made using a 19 G needle (Terumo, São Paulo, Brazil), and all threads were introduced into the same entry point regardless of the quantity used. Then, each thread was passed through the subcutaneous plane in a caudal direction toward the vicinity of the labiomandibular sulcus. The threads were carefully inserted, ensuring no resistance during the passage, with the endpoints of the threads positioned 1.5 cm apart for the group receiving 3 threads per hemiface and 1 cm apart for the group receiving 6 threads per hemiface (Video).

After the insertion and positioning of all threads, manual tissue traction in a cranial direction was performed on all threads simultaneously. To calibrate this traction in all patients, a tensiometer (Cremer, Paraná, Brazil) with a constant force of 150 N was always used. Finally, all patients were instructed to avoid physical exertion for 3 days and to refrain from making abrupt facial movements for 1 week.

### Stereophotogrammetry

Three-dimensional (3D) photographs were obtained to assess tissue displacement and volume change using a 3D stereophotogrammetry device (Quantificare, Sophia Antipolis, France). Then, the DermaPix Database software (Quantificare) automatically integrates the stereo images to produce a 3D reconstruction. The LifeViz application (Quantificare) is used for processing and managing these 3D images, allowing for the calculation and highlighting of volumetric differences and tissue displacement between the compared images.

**Figure 2.**
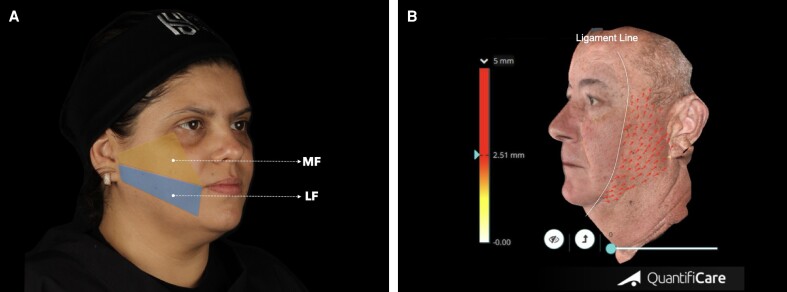
Schematic representation of the predefined middle face and lower face regions for (A) volumetric assessment in a 48-year-old female patient and (B) facial soft-tissue repositioning in a 53-year-old male patient using a stereophotogrammetry device. Arrows are pointing toward soft-tissue displacement direction.

Volumetric analyses were conducted at 3 specific time points: before treatment (T0), 20 days (T1), and 60 days (T2) after treatment. Volumetric changes were evaluated in 2 predefined regions, as shown in Figure 2A: the midface (MF), defined by the area bounded from the tragus to the outer corner of the eye, to the oral commissure, and back to the tragus; and the lower face (LF), defined by the area from the tragus to the oral commissure, to the mandibular margin, and back to the tragus.

Additionally, tissue displacement on the lateral face was assessed by counting the arrows automatically generated by the 3D software, which were configured to indicate displacements >2.5 mm, as per the software's adjustable threshold setting (Figure 2B).

### Global Aesthetic Improvement Scale

The patient and a blinded independent specialist, with at least 5 years of experience in facial aesthetic procedures, including treatments with PDO threads, were asked to rate the obtained results using the Global Aesthetic Improvement Scale (GAIS). The GAIS consists of a 5-point scale ranging from −2 to 2 (from worst to best), with −2 = “Very much worse,” −1 = “Somewhat worse,” 0 = “No change,” 1 = “Somewhat better,” and 2 = “Very much better.” GAIS results were assessed 60 days after the procedure, and baseline photographs were used by the volunteers and specialists (DDS, PhD, Spec., in facial aesthetics) to aid in remembering the before-treatment characteristics to precisely rate the results.

### Statistical Analysis

A total of 44 facial sides originating from 22 bilateral investigated patients were included in the analysis. All calculations were conducted using the Jamovi statistical program (The Jamovi Project, version 1.6.23). The results were considered statistically significant at a probability level of *P* < .05 to guide conclusions.

Volume change over time was obtained by subtracting the baseline volume from the after-treatment assessment (20 or 60 days: “T1—T0” and “T2—T0”). Repeated measures analysis of variance (ANOVA) was used to compare the results from volume changes and tissue displacement from the mid and lower lateral faces. A 1-way nonparametric ANOVA was used to assess the results from both GAISs (specialist and patients). Additionally, bivariate correlations (Pearson or Spearman, depending on the data format) were utilized to identify influences on the outcome parameters. Results are presented as mean value and the respective 1× standard deviation (SD) along with the data range—mean (SD) (range): independent of the data format to increase readability and understandability of the results presented.

## RESULTS

### Demographics

This study included 22 patients, of whom 18 were female (81.81%) and 4 were male (18.19%), with a mean age of 40.50 years (range, 31-57 years) for G1 and 43.90 years (range, 30-58 years) for G2 (*P* = .23). The mean BMI was 27.34 (±4.01) kg/m^2^, with no statistical differences between groups (*P* = .116): G1—26.50 (±4.42) kg/m^2^ and G2—28.40 (±3.26) kg/m^2^ (Table 1). The average follow-up time for patients was 65 days, with all patients completing follow-up within the range of 60 to 70 days posttreatment.

**Table 1.** Demographics

Demographics	Value
Participants	22
Male	4
Female	18
BMI	27.34 ± 4.01 (*P* = .116)
Age	43.90 ± 8.20 (*P* = .230)
Group 1 (6 PDO threads)	11
Male	2
Female	9
BMI	26.50 ± 4.42
Age	40.50 ± 6.60
Group 2 (12 PDO threads)	11
Male	2
Female	9
BMI	28.40 ± 3.26
Age	43.90 ± 9.60

PDO, polydioxanone.

### Volumetric Assessments

Regardless of the groups, significant volumetric changes were found over time (T1-T0 vs T2-T0) for MF (*P* = .015) and LF (*P* = .008) assessments. However, when intergroups comparisons were done, no significant volumetric differences were found at MF (*P* = .645) or LF (*P* = .925) along time.

**Figure 3.**
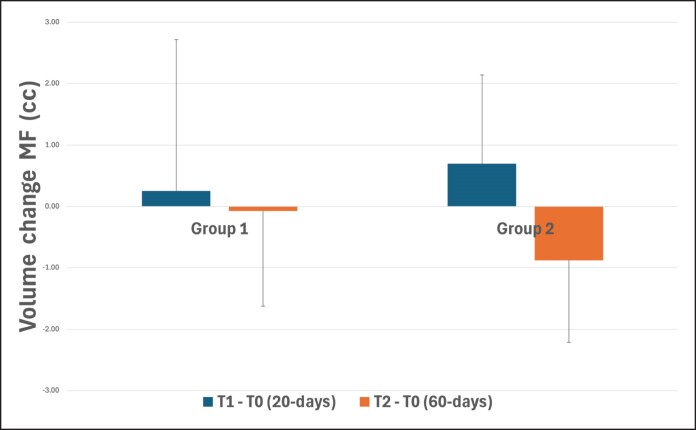
Assessment and comparison of volumetric changes between groups (Group 1—6 threads; Group 2—12 threads) at different timepoints in the middle face region. No significant differences were found between groups (*P* = .645). MF, midface.

In the MF, intragroup evaluation showed an increase of 0.25 (±2.46) cc for G1 and 0.7 (±1.44) cc for G2 after 20 days. Nonetheless, after 60 days, a volume decrease of −0.076 (±1.55) cc for G1 and −0.88 (±1.33) cc for G2 was observed when compared with baseline, as shown in Figure 3.

**Figure 4.**
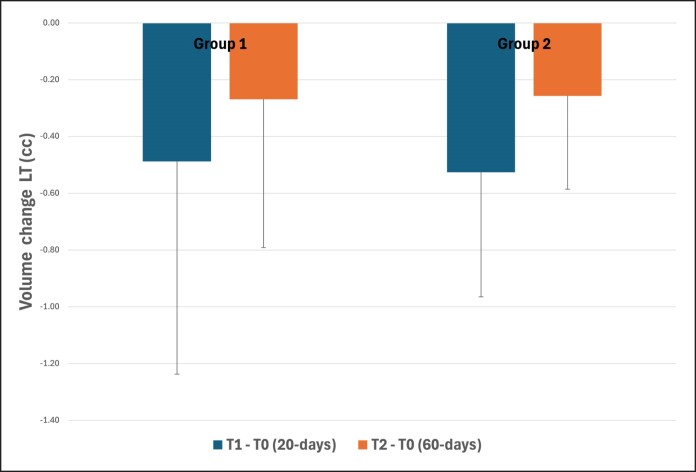
Assessment and comparison of volumetric changes between groups (Group 1—6 threads; Group 2—12 threads) at different time points in the lower face region. No significant differences were found between groups (*P* = .925).

In the LF, intragroup evaluation showed a decrease of −0.49 (±0.75) cc for G1 and −0.53 (±0.44) cc for G2 after 20 days. However, after 60 days, a volume decrease of −0.27 (±0.52) cc for G1 and −0.26 (±0.33) cc for G2 was observed when compared with baseline, as shown in Figure 4.

### Displacement Assessments

**Figure 5.**
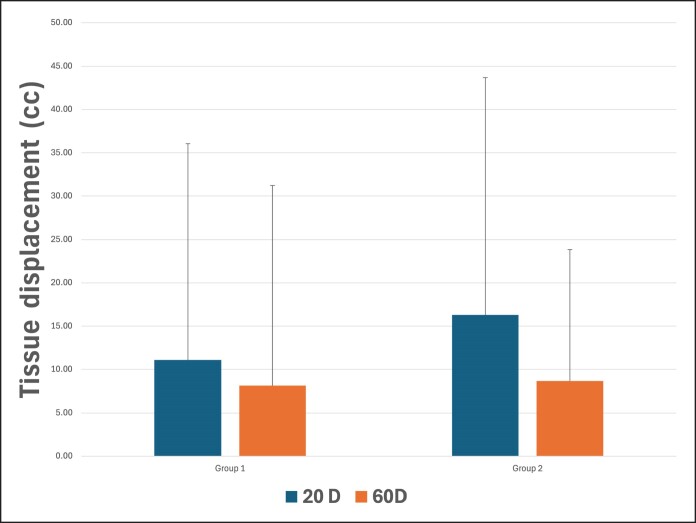
Assessment and comparison of tissue displacement between groups (Group 1—6 threads; Group 2—12 threads) at different time points. No significant differences were found between groups (*P* = .821).

Independently of the groups, tissue displacement showed statistically significant differences (*P* = .039) at the lateral face comparing 20 and 60 days. However, no significant differences were found between groups (*P* = .821) at 20 or 60 days. A mean tissue displacement of 11.10% (±25) and 8.13% (±23.08) was found for G1 at 20 and 60 days, respectively, and 16.30% (±27.39) and 8.65% (±15.20) were found for G2 (Figure 5).

### Global Aesthetic Improvement Scale Score

**Figure 6.**
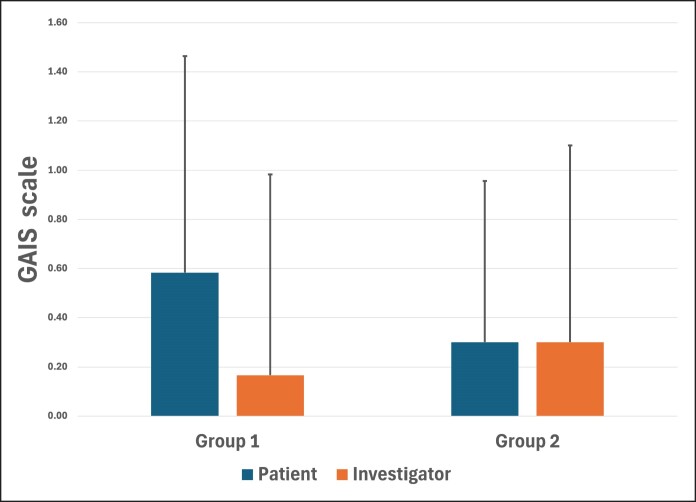
Subjective patient and investigator assessment using the Global Aesthetic Improvement Scale 60 days after treatment. No significant differences between groups (Group 1—6 threads; Group 2—12 threads) rated by the patient (*P* = .309) or the specialist (*P* = .558) were found. Also, no differences were found comparing both scores (patient vs investigator; *P* = .051).

GAIS scores were recorded for both groups at 60 days postprocedure (Figure 6). In G1 (6 PDO threads), the average GAIS score was 0.58 (±0.88) for the patient and 0.17 (±0.82) for the specialist. On the other hand, in G2 (12 PDO threads), mean GAIS score was 0.30 (±0.66) for the patient and 0.30 (±0.80) for the specialist. In both patient and specialist assessments, no statistically significant differences were observed between groups in terms of perceived improvement (*P* = .31 for patient and *P* = .56 for specialist). Finally, when comparing GAIS scores of patients vs specialists, no significant differences were found (*P* = .051).

### Adverse Events

Eleven patients reported discomfort related to pain at the 20 day follow-up. Of these, 5 were from G1 and 6 were from G2. The patients were medicated with an analgesic (Dipyrone 500 mg) and monitored. No other issues were reported, and all patients were free of discomfort by the 60 day follow-up.

## DISCUSSION

**Figure 7.**
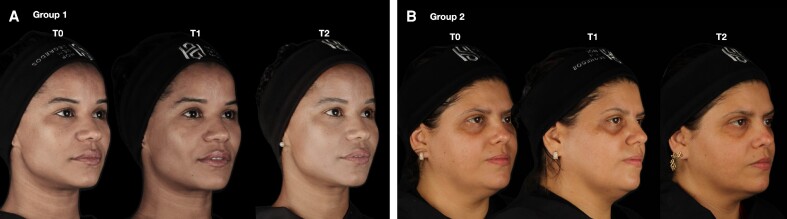
Photographic assessment of (A) a 38-year-old female patient from Group 1 and (B) a 47-year-old female patient from Group 2 at T0 (baseline), T1 (20 days), and T2 (60 days).

In this randomized, comparative clinical trial, the authors assessed the impact of the number of PDO threads on tissue displacement, volume changes, and patient satisfaction. Overall, no significant differences were found between the use of 6 threads (G1) and 12 threads (G2) across all observed parameters over time (Figure 7). These results suggest that the number of PDO threads may not significantly influence the sustained outcomes of lifting.

Volumetric changes in the MF and LF were evaluated over time in both groups. In the MF, a significant increase in volume was observed at the 20 day follow-up for both groups. Conversely, both groups exhibited a decrease in volume in the LF at the same time point. These findings support the concept of mechanical interaction between barbed suspension threads and the SMAS. The threads, inserted to firmly anchor the tissue, allow for tissue repositioning after cranial traction (ie, lifting effect), resulting in increased volume in the MF and decreased volume in the LF.^8,9^ Although not statistically significant, there was a tendency toward greater lifting effects with the use of more threads in the short term. These results rely on the threads’ ability to generate sufficient tension to mobilize and support facial soft tissues, promoting lifting, rejuvenated contours, and improvement of sagging without invasive surgical interventions.^10^

At the 60 day follow-up, the MF volume decreased compared with the 20 day follow-up, whereas the LF still showed a reduction in volume but in a lesser extent than at 20 days. These findings can be explained by biomechanical principles and tissue dynamics.^11^ The initial (20 days) cranial tissue displacement (lifting effect) depends entirely on the initial tension created by the PDO-thread traction. Subsequently, a caudal counter-displacement force driven by gravity, tissue weight, and facial mimics becomes more pronounced.^12,13^ This caudal counter-displacement may neutralize the initial lifting effect, leading to an outcome opposite to what was intended, because PDO threads are not anchored to a fixed structure.

Using a computer model of finite element analysis, Mousavi et al suggested that a greater number of threads and, consequently, a higher number of barbs can generate more traction, more tissue displacement, and greater volume.^14^ In addition, a previous study using fresh frozen specimen^15^ demonstrated the immediate lifting effects of facial threads, regardless of the technique or insertion angle. However, it was noted that this model does not accurately reflect the real-world effects, as support might also come from the formation of new collagen fibers—an aspect not observable in ex vivo models—as well as the influence of facial dynamics. Our results support these short-term findings. However, despite the initial (20 days) higher tissue displacement and lifting effect, these results were not sustained at a 60 day follow-up, regardless of the number of threads used per hemiface. The clinical model in our study suggests that PDO-thread mechanical traction may not be enough to maintain this tissue displacement, even when a higher number are used. Also, the production of new collagen fibers may not significantly affect lifting outcomes, at least at a 60 day follow-up. There is currently no standardization or clear understanding of PDO threads’ ability to stimulate collagen production, though some animal models have shown this benefit.^16^

Although the semiquantitative analysis revealed significant soft-tissue displacement over time (*P* = .039), the subjective measurements from the GAIS score showed no differences between patient (*P* = .31) and specialist (*P* = .56) ratings. This discrepancy may be attributed to the precision of the stereophotogrammetry device and software, which detects even minimal tissue displacements with a range of 0.1 to 5 mm, which may not be appreciated at first sight. Additionally, the lower GAIS scores in G2 compared with G1 may be a reflection of the more noticeable tissue ptosis in Group 2 at the 60 day follow-up, where the effects of the treatment may have been perceived as less durable, leading to a perception of diminished improvement by the patients.

It is important to note that this study was designed as a comparative analysis rather than a controlled study with a placebo or untreated group. The primary goal was to compare the effects of 2 different quantities of PDO threads on tissue displacement, volume changes, and patient satisfaction. Because the use of PDO threads is intended to cause lifting, it would not have been ethical or practical to include a control group that received no treatment or a placebo. Therefore, the comparison between the 2 different treatment protocols (6 vs 12 threads) was chosen as the focus of this investigation.

Importantly, no significant differences in BMI were found between groups at baseline (*P* = .116), indicating that initial volume differences did not influence the results. Moreover, despite the incidence of adverse events reported in the literature—such as asymmetry, bruising, swelling, discomfort, erythema, bleeding, visible threads, thread migration, infection, granuloma, skin rippling, skin irregularity, and thread extrusion^17^—our study reported minimal and transient adverse events. These low incidences of adverse events may be a consequence of the employed technique. The positioning of threads in a V-shaped configuration, as carried out in our investigation, creates a bidirectional tension that pulls the tissues toward the center and showed to be highly effective in lifting and supporting sagging tissues.^18,19^ Another important consideration is the possibility of using 1 or more insertion points for the cannula that carries the thread, with the option to finish the procedure with or without a knot between them. There is no defined standard in the literature regarding this practice. However, it is important to note that the absence of a knot at the end of the procedure can help avoid adverse events, such as thread palpation, migration, and possible infection.^20^

The strengths of this study include the objective evaluation using 3D surface scanning, which allows for precise measurement of skin movement and changes in surface volume projection between 2 3D images.^21^ Additionally, subjective assessments by the specialist, who had no direct involvement in the study, and by the patients themselves showed no significant differences between them or between groups. A limitation of this study is the limited follow-up points, with evaluations conducted at 20 and 60 days. This limits the understanding of the durability of the results and any potential late effects of collagen stimulation. Although the endpoint assessment showed no sustained lifting effects, these may persist or change over longer follow-up periods. Additionally, individual factors, such as variations in skin quality and lifestyle, were not detailed, which could influence the results and affect the generalizability of the conclusions. Future studies with more participants may provide a broader understanding of the long-term effects and variability in patient outcomes and should focus on optimizing thread placement and exploring the long-term biological effects, such as collagen stimulation, to enhance and sustain the outcomes of PDO-thread lifting procedures.

## CONCLUSIONS

This study demonstrates that although PDO threads can initially create tissue displacement and volume changes, the number of threads used does not significantly impact the longevity or magnitude of these effects. Initial changes in volume and tissue displacement diminished by 60 days in both cohorts, suggesting that, regardless of the number of threads placed, there is no long-term efficacy in terms of sustained tissue repositioning.

## Supplemental Material

This article contains supplemental material located online at https://doi.org/10.1093/asjof/ojaf002.

## Supplementary Material

ojaf002_Supplementary_Data
